# Depressive Symptoms Predict Major Depressive Disorder after 15 Years among Whites but Not Blacks

**DOI:** 10.3389/fpubh.2016.00013

**Published:** 2016-02-17

**Authors:** Ehsan Moazen-Zadeh, Shervin Assari

**Affiliations:** ^1^Medicine and Health Promotion Institute, Tehran, Iran; ^2^Mental Health Research Center, Tehran Psychiatric Institute, School of Behavioral Sciences and Mental Health, Iran University of Medical Sciences, Tehran, Iran; ^3^Department of Psychiatry, University of Michigan, Ann Arbor, MI, USA; ^4^Center for Research on Ethnicity, Culture and Health, School of Public Health, University of Michigan, Ann Arbor, MI, USA

**Keywords:** ethnic groups, African Americans, depressive symptoms, depression, validity, reliability

## Abstract

**Background:**

Black–White differences are shown in psychosocial and medical correlates of depressive symptoms and major depressive disorder (MDD). The current longitudinal study compared Blacks and Whites for the association between baseline depressive symptoms and subsequent risk of MDD after 15 years.

**Methods:**

Data were obtained from the Americans’ Changing Lives (ACL) Study that included 3,361 individuals (2,205 Whites and 1,156 Blacks) from 1986 to 2001. Baseline depressive symptoms measured using an 11-item Center for Epidemiological Studies-Depression (CES-D) in 1986 were predictors. The outcome of 12-month MDD was measured using the Composite International Diagnostic Interview (CIDI) in 2001. Covariates such as baseline socio-demographics (SES), financial difficulty, chronic medical conditions (CMC), and self-rated health (SRH) were measured in 1986. Logistic regression models were used to evaluate the association between baseline CES-D score and CIDI-based MDD after 15 years net of demographics, SES, CMC, and SRH. The models were applied in the pooled sample, as well as in Blacks and Whites. Data on reliability and factor structure of CES-D based on ethnicity were also reported.

**Results:**

In the pooled sample, we found an interaction between race and baseline depressive symptoms, suggesting a stronger effect of baseline depressive symptoms on the subsequent risk of MDD for Whites compared with that of Blacks. Such an interaction was significant net of socioeconomic and health status. Based on our ethnic-specific models, among Whites but not Blacks, baseline CES-D score was predictive of the subsequent risk of MDD after 15 years, net of SES and health at baseline. Black–White differences in the predictive role of CES-D scores on MDD could not be attributed to the ethnic differences in the reliability of the CES-D, which was even higher for Blacks compared with those of Whites. Loadings of the CES-D positive affect items were reverse among Blacks compared to Whites.

**Conclusion:**

Black–White differences exist in the association between baseline depressive symptoms and subsequent risk of MDD >15 years. Ethnic differences in the longitudinal link between baseline CES-D and subsequent risk of MDD may explain some of the Black–White differences in social, psychological, and medical correlates of depressive symptoms and depression. Future research is still needed to compare Blacks and Whites for factor structure of the CES-D.

## Introduction

More than 10% of global disability-adjusted life years – the principle index of global burden of diseases – are attributed to mental disorders (1), with major depressive disorder (MDD) accounting for the largest portion. Worldwide, MDD is among the four conditions with the highest contribution to years of life lost due to disability (2).

The Composite International Diagnostic Interview (CIDI) is a standard and well-respected survey tool commonly used in national mental health surveys. As the tool was developed for the World Mental Health project (3), the CIDI is a combination survey of the Diagnostic Interview Schedule and Present State Examination designed to be used by lay interviewers for the measurement of lifetime or recent DSM-IV-TR/ICD-10 mental disorders in large populations (3–8). The CIDI also serves as a measure for cross-cultural epidemiologic studies (3). CIDI yields valid results for Blacks with different ethnic groups (7, 8), as sensitivity and specificity of CIDI-based MDD are high for multiple ethnic groups (9).

In addition to MDD, as a clinical entity, the measurement of depressive symptoms also provides useful clinical information, as it reflects the severity of the mental health problem as well as the subsequent risk of MDD (2). The Center for Epidemiologic Studies Depression Scale (CES-D) is one of the most widely used survey instruments that are designed to measure the severity of depressive symptoms in epidemiological studies (10). Whether CES-D provides comparable results for Whites and Blacks in clinical and community settings has received a considerable scholarly attention (10–21).

It is still unknown whether self-reported measures of depressive symptoms can provide information concerning the subsequent risk of clinical MDD based on structural interview methods such as CIDI (22). Despite the fact that CES-D is one of the most widely used tools for the assessment of depressive symptoms among populations (14–16) and CIDI is one of the gold standard survey tools for determining the risk of MDD in epidemiological studies (23–25), the concordance between the two may vary across population groups (22, 23).

In the current study, we hypothesize that Blacks and Whites differ in the longitudinal association between baseline CES-D and subsequent risk of CIDI-based MDD in the United States. Initial support for this hypothesis comes from three bodies of evidence. First, the reliability of measures of depressive symptoms may be influenced by psychosocial factors as well as clinical conditions (26, 27), both factors are differently distributed in Blacks and Whites (28–32). Some studies imply that CES-D score may differently represent true depression in Whites and Blacks (33, 34). In addition, there have been inconsistent results comparing the reliability of CES-D scale across ethnic groups (11, 13, 35–37). Although some studies have found slightly a lower reliability for CES-D in Blacks (13, 35), there are other studies reporting a higher reliability for negative affect and interpersonal problem domains of CES-D in Blacks compared with those of Whites (11, 36, 37). This is particularly important given the role of ethnicity, culture, social class, and education on survey response style (38, 39).

Second, differences in the pattern of response to individual items have been observed between Whites and Blacks in the previous research (35). In fact, it is proposed that although latent factorial modeling of CES-D scale proves to be invariant across ethnicities in some studies (12, 13, 21, 35–37), primary items of CES-D might be group specific (12, 35), for example, Whites, especially White women, are less likely to respond positively to the item “people are unfriendly” (35). Blacks are more likely than Whites to endorse two interpersonal items (unfriendly and disliked), which may be a consequence of interpersonal racial discrimination or higher levels of interpersonal stress (40). Cultures are shown to be different in endorsement and meaning of positive and negative affect items (41).

Third, a growing literature is showing ethnic differences in magnitude and direction of the associations between depressive symptoms and clinical depression, including but not limited to socioeconomic status, chronic medical conditions (CMC), self-rated health (SRH), body mass index (BMI), and mortality (31, 32, 42–48). For instance, compared with Whites, Blacks have more CMC, higher mortality rate, and worse SRH, while they have lower frequency of clinical depression. Furthermore, Blacks and Whites differ in the associations between depression, medical conditions, behaviors, and well-being (42, 49). For instance, psychiatric disorders may have smaller effects on the perception of mental health among Blacks compared with Whites (42, 49). This new evidence suggests that the concordance between endorsement of criteria for psychiatric disorders and self-rated scales may differ across ethnic groups.

Although Blacks and Whites may potentially differ in their predictive role of CES-D on the subsequent risk of MDD based on CIDI, we are not aware of any previous studies investigating this issue. Therefore, we conducted this study to compare Blacks and Whites for the association between baseline depressive symptoms and subsequent risk of MDD in a 15-year cohort of American adults.

## Materials and Methods

### Design and Setting

We used the data obtained from the Americans’ Changing Lives (ACL) study. As a nationally representative cohort study, the ACL included U.S. adults aged ≥25 years from 1986 to 2011. Detailed methodology of the ACL study is documented elsewhere in the literature (50, 51).

### Sampling and Participants

Stratified multistage probability sampling was used in the ACL study. Baseline study population included 3,617 adults aged ≥25 years from non-institutionalized continental U.S. residents including Blacks and older adults (aged >60 years) who were oversampled at twice the rate of others. Our study is based on Whites and Blacks from wave 1 through wave 5 of the study: 1986, 1989, 1994, 2002, and 2011 (total *N* = 3,361; Whites = 2,205; Blacks = 1,156).

### Process

Baseline data on demographics (age, gender), socioeconomics, health status, and depressive symptoms were collected via face-to-face interview in wave 1. Our main predictor of interest was baseline depressive symptoms measured by the CES-D in 1986. Clinical MDD after 15 years was measured by CIDI, as our main outcome. Baseline demographic factors, socioeconomics status, and health status (CMC and SRH) were measured in order to account for potential confounding effects.

### Measures

#### Demographic Factors

We collected baseline data on gender (considering male as the reference) and age (in years) from the ACL database.

#### Socio-Demographics

We collected baseline data on education (years of schooling) and household income (as a continuous variable) from the ACL database.

#### Depressive Symptoms

The CES-D scale addresses major components of depressive symptomatology including: “depressed mood, feelings of guilt and worthlessness, feelings of helplessness and hopelessness” (10). The abbreviated version of the CES-D was used in the ACL study, which is composed of 10 items (16). Each item scores from 1 to 3 with the total score ranging from 10 to 30. We used *Z* scores instead of raw scores for between-group comparison. Validity and reliability of the abbreviated version of the CES-D are well documented (14–16).

#### Major Depressive Disorder

Composite International Diagnostic Interview of the World Mental Health project was used in the ACL study. The CIDI is a tool for the diagnosis of recent and lifetime mental disorders by trained interviewers (3). CIDI has shown a good concordance with Structured Clinical Interview for DSM-IV (SCID) diagnoses, especially MDD (4–6). Area under the receiver operating characteristic curve (AUC) has confirmed a high concordance between CIDI and SCID by psychiatrists in diagnosing mental disorders (3–5). In addition, optimal diagnostic thresholds of CIDI-SC have shown similar results with SCID in calculating the prevalence of mental disorders (6).

#### Chronic Medical Condition

In the ACL study, participants were asked to report about having one or more of the following seven focal conditions that were informed by a healthcare provider: hypertension, diabetes, heart disease, stroke, cancer, chronic lung disease, and arthritis. More details can be found elsewhere (31, 51).

#### Self-Rated Health

As a measure of an individual’s subjective health, SRH assesses responses to the question that “How would you rate your overall mental health?” based on the following five scales: poor, fair, good, very good, and excellent. Previous studies have used SRH in two ways both of which have shown acceptable validity and reliability (52–55), as a dichotomous measure with two categories of poor/fair and good/very good/excellent instead of the original five categories and as a continuous measure with the scores of 1 (excellent) to 5 (poor). We selected the SRH as a dichotomous measure in our study. As researches have shown that dichotomized SRH is also valid, several studies have treated this variable as a dichotomous variable [poor/fair vs. good/very good/excellent] (54, 55).

#### Ethnicity

The ACL study used multiple survey items in defining race and ethnicity of the participants at baseline. First, the participants had to answer an open-ended question: “In addition to being American, what do you think of as your ethnic background or origins?” Then, a multiple-choice question was asked with the possibility that multiple categories could be chosen: “Are you White, Black, American Indian, Asian, or another race?” In that manner, those who answered with more than one non-White group, had to say which category “best described” their race. The participants were also asked about their fathers’ last name and the state or country in which the participant, participant’s mother, and participant’s father were born. Finally, the participants had to answer whether they were of “Spanish or Hispanic descent, that is, Mexican, Mexican American, Chicano, Puerto Rican, Cuban, or Other Spanish?” Race categories were constructed based on the participants’ responses to the abovementioned questions: “Non-Hispanic White,” “Non-Hispanic Black,” “Non-Hispanic Native American,” “Non-Hispanic Asian,” and “Hispanic.” We only included non-Hispanic White and non-Hispanic Black individuals in our analysis.

### Ethics

Informed consent was obtained from the participants in the ACL study. The University of Michigan institutional review board has approved our study in accordance with the Code of Ethics of the World Medical Association (Declaration of Helsinki, Edinburgh 2000 revision).

### Statistical Analysis

We used Stata 13.0 (Stata Corp., College Station, TX, USA) as well as Statistical Package for Social Sciences v.20 (SPSS 20) for data analysis. SPSS was used for exploratory factor analysis and testing reliabilities. Stata was used for multivariable analysis. Stata enabled us to account for the complex sampling design of the ACL study by considering sampling and nonresponse weights. We used Taylor series linearization to estimate standard errors in subsamples. *P*-value of significance cutoff point was considered 0.05. *Z* scores were used instead of raw scores for the comparison of CES-D scale results among Blacks, Whites, and the pooled sample.

For bivariate analysis, we used independent-samples *t* test to compare CES-D scores between Blacks and Whites.

We used logistic regression models to evaluate the baseline CES-D scores as well as demographics, SES, CMC, and SRH in terms of their association with the diagnosis of CIDI-based MDD after 15 years. The models were applied in the pooled sample, as well as in Blacks and Whites.

We used Cronbach’s alpha to describe the reliability of the CES-D among Whites and Blacks. We also used inter-item correlation coefficients to determine the concurrent validity of the instrument by measuring the associations between baseline depressive symptoms overall score calculated through CES-D, each CES-D item, and diagnosis of depression after 15 years in 2001. The correlation analyses were carried out in the pooled sample, and for Blacks and Whites.

We also conducted an exploratory factor analysis using varimax rotation to compare the factor structure of the CES-D scale in the pooled sample, and for Whites and Blacks. As we did not fix the number of factors, the program suggested the following three factors: negative affect, positive affect, and interpersonal problems.

## Results

Table 1 presents descriptive statistics for CES-D *Z* score and each of the 11 items in the pooled sample, Whites, and Blacks. Independent-samples *t* test showed higher mean CES-D *Z* scores for Blacks than Whites (0.35 ± 1.12 vs. −0.03 ± 0.99, *P* < 0.01).

**Table 1:** **Descriptive statistics for baseline CES-D items based on ethnicity**.

	Mean (SD)	Mean (SD)	Mean (SD)

	All	Whites	Blacks
CES-D-11, *z* score, mean	0.10 (1.06)	−0.03 (0.99)	0.35 (1.12)
CES-D-11, raw score, mean	1.48 (0.43)	1.42 (0.41)	1.58 (0.45)
Item 1: I felt depressed	1.46 (0.10)	1.41 (0.10)	1.57 (0.20)
Item 2: everything was effort	1.61 (0.71)	1.52 (0.66)	1.79 (0.75)
Item 3: sleep was restless	1.67 (0.70)	1.67 (0.70)	1.67 (0.70)
Item 4: I was happy	1.19 (0.58)	1.18 (0.57)	1.21 (0.62)
Item 5: I felt lonely	1.43 (0.61)	1.37 (0.59)	1.53 (0.65)
Item 6: people were unfriendly	1.26 (0.51)	1.21 (0.46)	1.37 (0.58)
Item 7: I enjoyed life	1.16 (0.55)	1.16 (0.54)	1.17 (0.56)
Item 8: did not feel like eating	1.37 (0.60)	1.31 (0.57)	1.50 (0.64)
Item 9: I felt sad	1.43 (0.59)	1.39 (0.58)	1.50 (0.61)
Item 10: felt people dislike me	1.20 (0.46)	1.15 (0.40)	1.29 (0.54)
Item 11: I could not get going	1.56 (0.62)	1.53 (0.60)	1.60 (0.65)

*CES-D; Center for Epidemiologic Studies Depression scale*.

### Model in the Pooled Sample

Table 2 demonstrates the results of logistic regression models on the association between baseline depressive symptoms and CIDI-based MDD after 15 years in the pooled sample, as well as Whites and Blacks, net of baseline socio-demographic and health status. In the pooled sample, being Black did not have a main effect on the subsequent risk of MDD. In the pooled sample, higher CES-D scores (OR = 1.43, 95% CI = 1.14–1.80) at baseline were positively associated with the risk of CIDI-based MDD after 15 years. In addition, higher age (OR = 0.97, 95% CI = 0.95–0.99) and female gender (OR = 1.86, 95% CI = 1.23–2.82) were positively associated with the risk of CIDI-based MDD after 15 years. The association for the number of CMC (OR = 1.21, 95% CI = 0.98–1.49), chronic financial stress (OR = 0.98, 95% CI = 0.75–1.28), and education (OR = 1.02, 95% CI = 0.60–1.75) were nonsignificant. In the pooled sample, race and baseline depressive symptoms showed a significant interaction, suggesting a stronger effect of baseline depressive symptoms on subsequent MDD for Whites compared with those of Blacks. Such interaction was significant above and beyond socioeconomic and health factors (Table 2).

**Table 2:** **Summary of logistic regression models on the association between baseline CES-D scores and CIDI-based depression after 15 years based on ethnicity**.

	OR (95% CI)	OR (95% CI)	OR (95% CI)	OR (95% CI)	OR (95% CI)	OR (95% CI)	OR (95% CI)	OR (95% CI)	OR (95% CI)	OR (95% CI)	OR (95% CI)	OR (95% CI)

	All						Whites			Blacks		
Age	0.98 (0.96–1.00)^#^	0.98 (0.96–1.00)^#^	0.98 (0.96–1.00)^#^	0.98 (0.96–1.00)^#^	0.97 (0.95–0.99)**	0.97 (0.95–0.99)*	0.98 (0.96–1.00)^#^	0.98 (0.96–1.00)^#^	0.97 (0.95–0.99)*	0.98 (0.93–1.03)	0.97 (0.91–1.03)	0.98 (0.91–1.05)
Gender (female)	1.86 (1.22–2.82)**	1.84 (1.21–2.81)**	1.87 (1.23–2.83)**	1.85 (1.22–2.82)**	1.86 (1.23–2.82)**	1.85 (1.22–2.81)**	1.94 (1.25–3.01)**	1.95 (1.26–3.02)**	1.95 (1.26–3.01)**	0.97 (0.37–2.55)	0.99 (0.45–2.18)	0.91 (0.37–2.27)
Chronic financial stress	–	–	0.99 (0.76–1.30)	1.00 (0.77–1.30)	0.98 (0.75–1.28)	0.98 (0.76–1.28)	–	0.99 (0.75–1.31)	0.98 (0.74–1.29)	–	1.14 (0.66–1.96)	1.17 (0.33)
Education (>12 years)	–	–	0.88 (0.54–1.45)	0.89 (0.54–1.48)	1.02 (0.60–1.75)	1.02 (0.59–1.77)	–	0.92 (0.54–1.57)	1.07 (0.59–1.93)	–	0.66 (0.23–1.91)	0.58 (0.18–1.84)
Household income	–	–	1.01 (0.90–1.13)	1.01 (0.90–1.13)	1.02 (0.90–1.15)	1.02 (0.91–1.15)	–	1.00 (0.89–1.14)	1.01 (0.89–1.15)	–	1.06 (0.85–1.32)	1.07 (0.85–1.35)
Chronic medical conditions	–	–	–	–	1.21 (0.98–1.49)^#^	1.21 (0.98–1.49)^#^	–	–	1.28 (1.03–1.59)*	–	–	0.43 (0.18–1.00)*
Self-rated health (fair/poor)	–	–	–	–	1.65 (0.84–3.26)	1.63 (0.82–3.24)	–	–	1.50 (0.70–3.23)	–	–	4.32 (1.37–13.67)*
Race (Blacks)	0.64 (0.38–1.09)^#^	0.81 (0.47–1.39)	0.64 (0.37–1.10)	0.80 (0.46–1.39)	0.64 (0.37–1.11)	0.80 (0.46–1.38)	–	–		–	–	–
Depressive symptoms (CES-D)	1.52 (1.23–1.89)***	1.58 (1.25–1.99)***	1.52 (1.23–1.88)***	1.58 (1.26–1.98)***	1.43 (1.14–1.80)**	1.48 (1.16–1.90)**	1.58 (1.25–1.99)***	1.58 (1.25–1.98)***	1.48 (1.15–1.90)**	0.97 (0.64–1.46)	0.93 (0.65–1.33)	0.92 (0.64–1.34)
Depressive symptoms × Blacks	–	0.61 (0.38–0.96)*	–	0.61 (0.38–0.96)*	–	0.61 (0.38–0.97)*	–	–	–	–	–	–

^#^*P* < 0.1, *P < 0.05, **P < 0.01.

*CES-D, Center for Epidemiologic Studies Depression scale; CIDI, Composite International Diagnostic Interview*.

### Model among Whites

Similarly, in Whites, higher CES-D scores (OR = 1.48, 95% CI = 1.15–1.90) were significantly associated with CIDI-based MDD. Among Whites, higher age (OR = 0.97, 95% CI = 0.95–0.99), female gender (OR = 1.95, 95% CI = 1.26–3.01), higher number of CMC (OR = 1.28, 95% CI = 1.03–1.59), chronic financial stress (OR = 0.98, 95% CI = 0.74–1.29), and education (OR = 1.07, 95% CI = 0.59–1.93) were nonsignificant (Table 2).

### Model among Blacks

Different from the pooled sample and Whites, CES-D scores (OR = 0.92, 95% CI = 0.64–1.34) showed a nonsignificant and negative association with the subsequent risk of CIDI-based MDD. Among Blacks, the baseline SRH strongly predicted CIDI-based depression (OR = 4.32, 95% CI = 1.37–13.67) suggesting larger OR compared with any other covariates. For Blacks, gender (OR = 0.91, 95% CI = 0.37–2.27), education (OR = 0.58, 95% CI = 0.18–1.84), chronic financial stress (OR = 1.17, 95% CI = 0.66–2.06), and CMC (OR = 0.43, 95% CI = 0.18–1.00) were not significant predictors of subsequent MDD risk, a pattern which was different from the pooled sample and Whites (Table 2).

### Inter-Item Correlations

Tables 3 and 4 present correlation coefficients between baseline total CES-D score, CES-D items, and CIDI-based MDD after 15 years in the pooled sample and each ethnic groups. In the pooled sample, the absolute value of correlation coefficients between overall CES-D scores and item-specific scores ranged from 0.257 to 0.733 with most coefficients being above 0.50. The coefficients for inter-item associations ranged from 0.640 and 0.582 with most coefficients ranging from 0.200 to 0.400. In the case of the association of overall CES-D score and item-specific scores with the diagnosis of depression through CIDI for the past 12 months after 15 years, the absolute value of the coefficients ranged from 0.058 to 0.164. Among Whites, the absolute value of the coefficients for the association between CES-D and item-specific scores ranged from 0.241 to 0.732 (most of the coefficients were above 0.500), for inter-item association ranged from 0.200 to 0.591 (most of the coefficients were between 0.200 and 0.400), and for the association of CES-D and item scores with the diagnosed depression through CIDI ranged from 0.800 to 0.212. Among Blacks, correlation coefficients for the association between CES-D and item-specific scores showed values between 0.289 and 0.731 (most of the coefficients were above 0.500), 0.032–0.557 for inter-item associations (most of the coefficients were between 0.200 and 0.400), and 0.005–0.096 (all the values were below 0.100) for the association of CES-D and items with the CIDI-based MDD.

**Table 3:** **Correlations between baseline CES-D score, CES-D items, and CIDI-based depression after 15 years in the pooled sample**.

		1	2	3	4	5	6	7	8	9	10	11	12	13
1	CES-D-11 *z* score, mean	1	0.733**	0.597**	0.543**	−0.624**	0.687**	0.512**	−0.562**	0.549**	0.733**	0.568**	0.596**	0.164**
2	I felt depressed		1	0.407**	0.344**	−0.439**	0.502**	0.273**	−0.365**	0.316**	0.582**	0.313**	0.378**	0.137**
3	Everything was effort			1	0.278**	−0.242**	0.319**	0.223**	−0.232**	0.317**	0.353**	0.264**	0.392**	0.072**
4	Sleep was restless				1	−0.237**	0.309**	0.187**	−0.185**	0.278**	0.334**	0.198**	0.324**	0.117**
5	I was happy					1	−0.375**	−0.198**	0.545**	−0.224**	−0.435**	−0.240**	−0.257**	−0.121**
6	I felt lonely						1	0.286**	−0.322**	0.323**	0.528**	0.297**	0.329**	0.093**
7	People were unfriendly							1	−0.160**	0.191**	0.274**	0.482**	0.170**	0.093**
8	I enjoyed life								1	−0.190**	−0.358**	−0.216**	−0.211**	−0.087**
9	Did not feel like eating									1	0.316**	0.196**	0.315**	0.058*
10	I felt sad										1	0.346**	0.390**	0.154**
11	Felt people dislike me											1	0.231**	0.098**
12	I could not get going												1	0.079**
13	CIDI 12 months MDD													1

*CES-D, Center for Epidemiologic Studies Depression scale; CIDI, Composite International Diagnostic Interview*.

**Table 4:** **Unadjusted correlations between baseline CES-D total score, CES-D items, and CIDI-based MDD after 15 years based on ethnicity**.

		1	2	3	4	5	6	7	8	9	10	11	12	13
1	CESD-11 *z* score, mean	1	0.732**	0.611**	0.534**	−0.645**	0.661**	0.471**	−0.586**	0.518**	0.732**	0.531**	0.588**	0.212**
2	I felt depressed	0.720**	1	0.421**	0.318**	−0.446**	0.492**	0.238**	−0.376**	0.292**	0.591**	0.291**	0.364**	0.159**
3	Everything was effort	0.543**	0.349**	1	0.272**	−0.257**	0.304**	0.206**	−0.254**	0.323**	0.357**	0.265**	0.417**	0.124**
4	Sleep was restless	0.582**	0.397**	0.302**	1	−0.237**	0.275**	0.162**	−0.191**	0.257**	0.324**	0.175**	0.315**	0.137**
5	I was happy	−0.590**	−0.418**	−0.196**	−0.237**	1	−0.365**	−0.184**	0.562**	−0.218**	−0.452**	−0.238**	−0.268**	−0.148**
6	I felt lonely	0.712**	0.496**	0.302**	0.375**	−0.380**	1	0.248**	−0.329**	0.287**	0.509**	0.240**	0.295**	0.110**
7	People were unfriendly	0.541**	0.287**	0.195**	0.231**	−0.204**	0.308**	1	−0.152**	0.146**	0.252**	0.467**	0.138**	0.136**
8	I enjoyed life	−0.528**	−0.340**	−0.187**	−0.174**	0.510**	−0.303**	−0.163**	1	−0.177**	−0.374**	−0.228**	−0.205**	−0.107**
9	Did not feel like eating	0.566**	0.319**	0.263**	0.322**	−0.216**	0.347**	0.209**	−0.201**	1	0.285**	0.136**	0.293**	0.080**
10	I felt sad	0.731**	0.557**	0.325**	0.356**	−0.396**	0.547**	0.287**	−0.325**	0.344**	1	0.321**	0.360**	0.194**
11	Felt people dislike me	0.596**	0.315**	0.219**	0.240**	−0.235**	0.346**	0.476**	−0.195**	0.233**	0.368**	1	0.217**	0.134**
12	I could not get going	0.609**	0.393**	0.346**	0.341**	−0.231**	0.372**	0.202**	−0.216**	0.337**	0.434**	0.241**	1	0.092**
13	CIDI 12 months MDD	0.055	0.096	−0.046	0.045	−0.053	0.066	0.007	−0.036	0.005	0.038	0.032	0.045	1

*P < 0.05, **P < 0.01, ***P < 0.001.

*Whites, up diagonal; Blacks, low diagonal*.

*CES-D, Center for Epidemiologic Studies Depression scale; CIDI, Composite International Diagnostic Interview; MDD, Major Depressive Disorder*.

### CES-D Factor Structure

Our factor analysis suggested three factor models (i.e., negative affect, positive affect, and interpersonal problems) in the pooled sample, Whites, and Blacks. Table 5 shows the results of factor analysis in the pooled sample and also in ethnic groups. Our factor analysis revealed two Black–White differences: (1) Item “Felt people disliked me” was better loaded with negative affect for Blacks; however, this item was better loaded with interpersonal problems factor for Whites. (2) The positive affect items including “I was happy” and “I enjoyed life” was loaded negatively on Factor 2 (negative affect) for Whites but positively for Blacks (Table 5).

**Table 5:** **Factor structure of CES-D based on ethnicity at baseline**.

		Positive affect	Negative affect	Interpersonal problems	Positive affect	Negative affect	Interpersonal problems	Positive affect	Negative affect	Interpersonal problems
				
		All			Whites			Blacks		
1	I felt depressed	−0.016	**0.733**	−0.076	0.024	**0.720**	−0.088	−0.006	**0.743**	−0.065
2	Everything was effort	−0.268	**0.568**	−0.113	0.262	**0.574**	−0.044	−0.302	**0.527**	−0.221
3	Sleep was restless	−0.330	**0.477**	−0.217	0.359	**0.429**	−0.242	−0.354	**0.580**	−0.088
4	I was happy	**0.676**	0.541	0.005	−**0.656**	0.548	−0.061	**0.683**	0.546	0.047
5	I felt lonely	−0.004	**0.686**	−0.070	0.008	**0.669**	−0.097	−0.012	**0.704**	−0.035
6	People were unfriendly	−0.228	0.454	**0.692**	0.165	0.409	**0.727**	−0.247	0.486	**0.660**
7	I enjoyed life	**0.717**	0.462	−0.054	−**0.691**	0.485	−0.096	**0.723**	0.458	−0.058
8	Did not feel like eating	−0.177	**0.530**	−0.321	0.234	**0.507**	−0.253	−0.106	**0.538**	−0.417
9	I felt sad	0.029	**0.730**	−0.048	−0.004	**0.718**	−0.061	0.052	**0.749**	−0.020
10	Felt people dislike me	−0.071	0.576	**0.594**	0.015	0.531	**0.631**	−0.069	0.612	**0.547**
11	I could not get going	−0.275	**0.564**	−0.279	0.328	**0.535**	−0.230	−0.239	**0.609**	−0.286

*CES-D; Center for Epidemiologic Studies Depression scale*.

*Extraction method: principal component analysis*.

*Bold numbers show items that are loaded in each factor*.

### CES-D Reliability

Reliability of CES-D was 0.793 among all, 0.773 among Whites, and 0.816 among Blacks.

## Discussion

We found that baseline CES-D score predicted the subsequent risk of CIDI-based MDD after 15 years in Whites but not in Blacks. This finding was not due to the reliability or factor structure of CES-D. We found a higher reliability of CES-D among Blacks compared with that of Whites. Similarly, among Whites and Blacks, CES-D was composed of three factors, namely negative affect, positive affect, and interpersonal problems.

Our results are in line with the previous findings on ethnic variations in the concordance between CIDI-based MDD and perception of mental health (42, 49, 56, 57). Ethnicity may change how particular psychiatric disorders such as MDD are reflected by scales that measure perceived mental health distress (56). The relationship between perceived mental health and psychiatric disorders may be weaker for non-Hispanic Blacks and other minority groups compared with that of non-Hispanic Whites (49). Even among Blacks, ethnicity may change based on how overall perceived mental health reflects clinical disorders based on DSM criteria (56, 57). The concordance between CES-D score – as a less specific measure of psychological distress – and clinical MDD based on DSM may depend on contextual factors such as ethnicity, culture, and social class (28).

In a study, Kim et al. found that mental SRH better reflects CIDI-based depression and anxiety disorders for Whites than Blacks (49). Interestingly, they found that a larger portion of Blacks rate themselves as poor/fair on mental SRH in comparison with Whites, and they less frequently endorse DSM-based diagnosis of depressive and anxiety disorders (49). This finding is replicated in our study where Blacks had higher scores on the CES-D scale but a lower prevalence of clinical depression according to CIDI after 15 years. These findings call into question the cross-ethnic equivalence of the CES-D scale as well as CIDI.

We found a higher reliability of CES-D scale in Blacks compared with that of Whites. Previous evidence has shown differences in the reliability of CES-D among ethnic groups using Cronbach’s alpha; however, the results are not consistent. In some studies, slightly lower reliability has been reported for CES-D in Blacks compared with that of Whites (13, 35). In other studies that have reported reliability coefficients for negative affect items and positive affect items separately, the reliability of items were inconsistently different between Blacks and Whites (11, 36, 37). Considering that depressive symptoms are subject to variations under different psychosocial and clinical situations, both of which are different in Blacks and Whites (26–32), reliability analyses of CES-D scale may have an implication that depressive symptoms are represented differently in Blacks and Whites (33, 34), whether due to the differences in their living conditions or item-specific differences in the responses given by each ethnic group (35). In the later case, it seems that the endorsement rate of certain CES-D items can be different between Whites and Blacks, for example, Whites, especially White women, less likely respond positively to the item “people are unfriendly” (35).

Our findings regarding exploratory factor analysis of CES-D scale among Blacks and Whites should be confirmed using confirmatory factor analysis. Our preliminary results were suggestive of some Black–White variations, for instance, the item “Felt people disliked me” was better loaded with negative affect for Blacks; however, this item was better loaded with the interpersonal problems factor for Whites. The reversed positive affect items including “I was happy” and “I enjoyed life” were loaded negatively on Factor 2 (negative affect) for Whites but positively for Blacks. These are in line with the results of previous research comparing low-socioeconomic Blacks with nationally representative samples (21). Scrutinizing age and gender-specific groups of Blacks and Whites have also yielded comparable results considering interpersonal problem domain and negative affect domain (35). On the other hand, other studies have shown factorial invariance and equality between samples of Black and White girls (37). Numerous studies on factor structure of 20-item CES-D scale have proposed four-factor models with high overall fits for both Blacks and Whites (21, 35–37); however, differences have been shown in the factor loadings between Blacks and Whites with interpersonal items having higher loadings in Blacks (21, 35). In a relatively recent meta-analysis, Kim et al. found that the four-factor models may not be suitable for application in all ethnic groups (58). However, they found inconsistency in the results of the studies reviewed. These findings are in accordance with our design of the statistical analysis focused on item-level differences of CES-D in Blacks and Whites, rather than latent factorial differences. We do not know whether Blacks and White differ mainly in the item or factor levels of CES-D scale. Further research is recommended to explore this issue, as ethnic variations in factor structure of measures that capture depressive symptoms may have implications for better understanding some of the Black–White differences in the social and medical correlates of depressive symptoms in the literature (12, 13, 42).

Based on our findings, Whites had lower CES-D scores despite higher rates of meeting criteria for CIDI-based MDD after several years. Literature has shown that Blacks have weaker association between CES-D scores and perceived mental health distress in comparison with Whites (42). It is not clear, however, if this is due to a different presentation of depressive symptoms and clinical depression in Blacks compared with Whites (33, 34). Previous research has shown that there may be differences in the perception of interpersonal interactions between Blacks and Whites (20, 59, 60) which in turn contributes to the variations in CES-D scores. Furthermore, it has been suggested that depressive symptoms differ in their importance for indicating the same underlying disorder (61).

We found higher correlation coefficients between CES-D items and subsequent risk of endorsement of criteria for MDD diagnosis based on CIDI among Whites compared with those of Blacks. We also found some preliminary differences in patterns of correlations between CES-D items between Whites and Blacks. These preliminary findings warrant for research on the factor analysis of CES-D scale in Blacks and Whites (11, 34–36).

There are a number of limitations in our study that should be mentioned. First, in our analysis, we used baseline depressive symptoms, CMC, and SRH although these constructs are subject to change over time. Second, we did not have a control for antidepressant medication that may confound or mediate differential effects associated with ethnicity observed in this study. Our results on factor analysis among Blacks and Whites were only preliminary. This warrants future research using a more robust approach. One solution is to compare the fit of items and subscales of CES-D between Blacks and Whites using confirmatory factor analysis. It is noteworthy that previous population studies concerning the CES-D scale have been subject to most of the limitations mentioned earlier. Up to our knowledge, this is the first study on the ethnic differences in the association between baseline depressive symptoms measured by CES-D scale and subsequent risk of MDD diagnosis based on CIDI after 15 years in a cohort of several thousand American adults. Most previous research related to our work has used cross-sectional designs (42, 49, 56). By controlling for education, income, financial stress, and physical health, we ruled out the confounding effect of socioeconomic status and health on ethnic differences in the predictive role of CES-D score on the subsequent risk of MDD.

In conclusion, baseline CES-D may be a weaker tool in predicting the subsequent risk of MDD in Blacks compared with Whites. Ethnic difference in concordance between baseline CES-D and subsequent risk of CIDI-based MDD helps us better understand the complex links between race, ethnicity, socioeconomics, depressive symptoms, depression, health, and mortality. Our findings may explain some of the racial and ethnic differences in psychosocial and medical correlates of depressive symptoms and MDD. Future research is needed to compare factor structure of the CES-D scale between Blacks and Whites.

### Compliance with Ethical Standards/Informed Consent

All procedures followed were in accordance with the ethical standards of the responsible committee on human experimentation (institutional and national) with the Declaration of Helsinki of 1975, and it was revised in 2000. Informed consent was obtained from all participants included in the study.

### Animal Studies

No animal studies were carried out by the authors for this article.

## Author Contributions

EM performed a comprehensive literature review and provided the first draft of the manuscript. SA was responsible for the design and analysis of the data. Both authors revised the manuscript and approved the final draft.

## Conflict of Interest Statement

The authors declare that the research was conducted in the absence of any commercial or financial relationships that could be construed as a potential conflict of interest.
